# Delusional Parasitosis or Morgellons Disease: A Case of an Overlap Syndrome

**DOI:** 10.1155/2023/3268220

**Published:** 2023-04-27

**Authors:** Fatmah Alhendi, Abdullatif Burahmah

**Affiliations:** Department of Oral Medicine, Ministry of Health of Kuwait, P. O. Box (5), Safat 13001, Kuwait

## Abstract

*Background.* Delusional parasitosis (DP) is a monosymptomatic hypochondriacal psychosis where the patient has the delusion of being infested with parasites, whereas Morgellons disease (MD) is described when the patient has fixed ideation of fibers or other materials emerging from skin. Both psychological and organic causes can result into the delusion of infestation, and careful examination is required to exclude secondary causes. Oral DP can result in self-inflected mutilations of the oral mucosa. To our knowledge, oral DP is only rarely reported in the literature. Here, we describe and discuss the management of a case of overlap between oral DP and oral MD at the oral medicine (OM) clinic. *Case Report.* A 50-year-old male presented to the OM clinic with ulcerations of oral and perioral tissues. Patient reported inflicting wounds to himself using a shaving blade to extirpate worms and pieces of glass from underneath his oral and perioral mucosa. Clinical and laboratory investigations ruled out parasitic infestations. Self-inflected ulcers were treated with topical steroids and prophylactic antifungals, and the patient was referred for psychiatric evaluation. A diagnosis of primary DP was reached, and the patient was managed with antipsychotics. *Practical Implications.* Oral health care providers should be familiar with oral manifestations of psychiatric disorders and should be able to manage such patients in a multidisciplinary team of internist, dermatologist, and psychiatrist.

## 1. Introduction

Parasitosis is any illness that is caused by a parasite, an organism that lives in or on another organism. Parasites that infect human include protozoans and helminths. They are transmitted to their hosts through the ingestion of contaminated food or water or through the bite of a fly or a tick [1]. Recently, infectious diseases including parasitic infections have been increasing in refugee and asylum-seeking individuals to high-income countries [2]. On the other hand, delusions of parasitosis, delusional parasitosis (DP), or Ekbom syndrome are all terms used to describe a type of hypochondrial psychosis where patients have a false believe that they are infested with parasites with the sensation of crawling or itchiness caused by the parasite moving under their skin [3]. This fixed believe causes these individuals to inflict injuries and causes ulcerations on their skin to remove the parasite [4]. Morgellons disease (MD), another psycho-dermatologic condition, refers to the false sensation of fibers, filaments, or even crystal-like materials emerging from non-healing wounds that these individuals create on the skin in an attempt to remove these disturbing, itchy, or painful objects [5]. DP is more prevalent in middle-aged women [6], and about 80% of patients have a comorbid psychiatric conditions, mainly depression [7, 8]. DP can be either primary, where there are no other psychiatric or organic causes, or secondary, due to co-existing psychiatric conditions, such as depression and schizophrenia, or due to organic causes, such as anemia, diabetes, infections, hypothyroidism, or cocaine abuse among others [9, 10]. Secondary, or organic, causes of the itching sensation should be ruled out by thorough examination and investigations to reach the diagnosis of primary DP. Individuals with DP exhibit a common and classical behavior of bringing samples of the material they collected from the skin as a proof of the infestation. This behavior often referred to as the “matchbox sign,” “Ziploc bag sign,” or “specimen sign” [11]. The etiopathophysiology of DP is still unknown, but an increase of extracellular dopamine level caused by malfunction of striatal dopamine transporter was proposed as the mechanism of action [12], which can be the cause of both primary and secondary forms of DP [11]. Management of DP can be pharmacologically, non-pharmacologically, or combination approaches. Antipsychotics are mainstay of treatment for DP, with risperidone being the first line due to its lower rate of extrapyramidal symptoms [13]. Pimozide, aripiprazole, and olanzapine are all alternative antipsychotics with variable efficacy and safety profiles [13]. Non-pharmacological therapy in the form of cognitive behavioral therapy (CBT) can be helpful as first-line management in patients with mild depression associated with DP [11]. Interestingly, psychotherapy alone seems to be effective in only 10% of patients diagnosed with DP [14]. Patients with severe forms of DP have a high risk of committing suicide and should be closely monitored [15]. Prognoses of DP is variable depending on the classification of DP. Patients with the primary type of DP and those secondary to medical conditions often have chronic course and less favorable prognosis than those diagnosed with drug-related DP [16]. If symptoms of DP manifest in the orofacial region, patient will often seek oral health care providers (OHCP) consultation who should be able to recognize the condition, make the appropriate referrals, and work closely with other health care specialists to optimize patient care. Hence, the purpose of this case report is to highlights the importance of being familiar with psychiatric conditions that may present as orofacial pain to avoid unnecessary procedures and treatments.

## 2. Case Report

**Figure 1:**
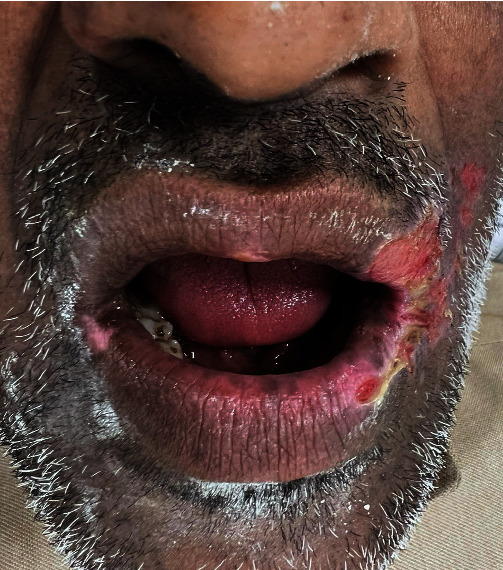
The patient presenting with oral and perioral ulcerations and extending to the facial skin.

A 50-year-old Caucasian male patient was referred from the oral surgery department to the oral medicine (OM) clinic for evaluation of oral and perioral ulcers as shown in Figure 1. While interviewing the patient, he seemed well kept, but stressed and anxious. The patient complained about the presence of impeded glass under his oral and perioral ulcers with a crawling sensation. In addition, he reported that his oral cavity is infested with worms and that the crawling sensation and pain being relieved after inflicting wounds and inducing bleeding using a sewing needle and a razor blade to extract the crawling worms.

The initial onset of this complaint started 1 month before, when the patient chewed tobacco in an attempt to quit smoking. Chewing tobacco ca. used a burning sensation that improved after cessation of smokeless tobacco usage. A week later, the patient experienced a burning and crawling sensation at the tip of the tongue after drinking cold water. When the patient checked his tongue, he saw small papules, which he purposely injured to induce bleeding. He did the same for the papules at oral and perioral regions whenever he felt the crawling and the foreign body sensations. This issue affected the patient's daily life as he could not sleep at night and became socially isolated. He lost 3 kg due to decreased food intake caused by the oral and perioral ulcers. The patient brought with him a bag with folded papers filled with clotted blood and remnants of soft tissues, which he considered dead worms extirpated from his wounds. The patient denied any recent travels or contact with sick individuals with a negative family history of similar problems or any psychiatric disorders. Moreover, he denied the use of any recreational drugs, over the counter medications, or herbal medications. He smokes 21 cigarettes per day for the past 30 years and has a family history of diabetes mellitus type 2. When interviewing him, he denied experiencing any audible or visual hallucinations, or having thoughts of harming others. The patient stated that he became depressed after he lost his son two years ago, and his lifestyle and personality changed dramatically afterwards, and only at that time, he had a strong thought of ending his life and he experienced it only once. The review of systems was otherwise unremarkable.

Patient was alert, vital signs, weight, height, and body mass index were within normal limits. Extraoral examination ruled out any facial asymmetry, lymphadenopathy, salivary gland enlargement, thyromegaly, cranial nerve abnormalities, or temporomandibular dysfunction. Skin at the commissures had ulcerations extending to the lower lip bilaterally with no signs of any parasites or foreign bodies. Intraorally, lower anterior gingival margins were with erosions and erythema. Generalized smoker's keratosis with melanosis were noted. Dentition was stable, without gross decay, and the tongue was devoid of any ulcers, masses, or lesions. Blood tests were requested including complete blood counts (CBC)/differentials, full biochemistry profile (liver function test and renal function test), thyroid stimulating hormone (TSH), vitamin B12, and glycosylated hemoglobin (Hb A1C) levels to exclude organic causes. Photographs were taken, and the patient consented to use his photographs for medical and educational purposes. Because the patient admitted that his wounds were self-inflected, the cause of his oral and perioral lesions were diagnosed to be traumatic in nature. A topical cream of 0.1% dexamethasone, chlorhexidine 1.15%, and nystatin 100,000 IU/g were prescribed to be applied three times daily for two weeks and to avoid irritating the wounds. In addition, patient was advised to seek smoking cessation consultation. To rule out psychogenic cause of patient's symptoms, he was referred to the psychiatric clinic for consultation. The patient was asked to return to the office after two weeks for re-evaluation or before that if his condition worsened or did not improve.

On his first follow-up, the patient noted a moderate improvement of his ulcers when using the topical cream. In addition, the patient starts to inflict wounds on the lower labial mucosa causing a continuous painful sensation that disturbed his sleep and affected his personal and oral hygiene. Systemic non-steroidal antiinflammatory drug (NSAID), diclofenac 50 mg once daily as needed, has been prescribed to alleviate the continuous pain, and he was advised to return to the office after two days to review his blood test results.

On the second follow-up, the patient's pain was relieved with NSAID. However, he started experiencing dizziness and lethargy, which might be due to constant bleeding from wounds, and he started to inflict wounds on his left index finger to extirpate what he thought were worms. On reviewing patient blood tests, they were within normal limits except for a slight decrease in vitamin B12. All the blood test results suggested volume loss and an imbalanced diet; therefore, organic causes for the patient's condition have been excluded. At that point, the psychiatric clinic contacted the OM clinic regarding this patient, and they confirmed the diagnosis of primary DP. As per the patient's psychiatrist, the patient was anxious when first presented to the psychiatry clinic and thought that this referral was not related to his oral pain. The treating psychiatrist revealed that the patient did not have any other psychiatric conditions or any family history of mental disorders after assessment at the psychiatric hospital. Olanzapine 10 mg was prescribed with significant improvement of oral and extraoral symptoms within one month. The patient returned to the OM clinic for a follow-up two months later with resolution of his symptoms except for esthetic complaints regarding the scarring and hypopigmentation at the site of perioral wounds. Patient was advised to seek dermatological consultation. The patient is still under both psychiatric and OM care.

## 3. Discussion

The present case report describes the clinical presentation and management of oral DP by OM specialists as part of multidisciplinary team of dermatologist, psychologist, hematologist, and infectious disease specialists (Table 1). Only few cases of oral delusional complaint reported in the literature (Table 2). The first case reported in the literature was of 76-year-old male who was referred to the Psychiatry Department of Kobe University School of Medicine, Japan, with a fixed believe that threads are emanating from the gingiva between his teeth and that these threads will later convert into worms for 3 months duration [17]. One significant part of his medical history was a left-side cerebral infarct that he suffered from 11 years ago, but without any residual symptoms [17]. Psychological, hematological, pathological, metabolic, and infectious etiologies were ruled out, but brain magnetic resonance imaging (MRI) was significant for old cerebral infarct in the right putamen [17]. Fortunately, patient delusions were resolved with antipsychotics [17]. A case was reported by Hanihara et al., about a 64-year-old female complaining of live worms infesting her mouth and moving around and to the back of her teeth [18]. Although the patient did not have previous history of substance abuse, metabolic, hematological, or psychiatric illness, she had a history of stroke revealed by computed tomography (CT) of the brain as hematoma in the left posterior thalamic region [18]. The administration of sulpiride, atypical antipsychotic, resulted in complete resolution of her oral symptoms [18]. Another case, being reported in 2010, when a 61-year-old Caucasian female presented to her periodontist with oral lesions for a duration of 2 years and a complaint of “numerous fibers” emerging from her oral lesion with itching and irritation from the lesion [19]. Pathological examination of these fibers revealed nothing, but synthetic fibers that were possibly implanted into the oral mucosa by the patient [19]. This case challenged the idea that MD is a form of DP and should be treated as such [19]. Another case was of 39-year-old woman presented to the OM clinic complaining of hair and insects coming out of her maxillary gingiva with oral tickling sensation and gingival bleeding for a period of 3 years [20]. Detailed extra and intraoral examination was unremarkable, but the patient does have a previous diagnosis of MD, bipolar disorder, and anxiety and was on medications by psychiatrist, but she discontinued using them [20]. This case is like our case, in this report, in that there is an overlap between symptoms of DP and MD. When the patient seeks OHCP to alleviate his/her symptoms, OHCP should exclude all secondary causes of DP and the patient should be carefully approached and correctly directed to other health care professionals as these patients have a fixed believe that their symptoms are real and caused by infestation of parasites. Any confrontation with the patient may lead to loss of rapport and failure to comply and follow up. One of the most difficult challenges we faced in our case is to introduce the psychological etiology as the cause of patient's symptoms and how to discuss with the patient the need to include psychiatrist in our management. Another issue we had is the need to subject the patient to a wide series of investigations and laboratory requests to exclude secondary causes of DP. While laboratory investigations were within normal limits, and there was no history of substance abuse, radiological and psychological assessment are still needed to rule out intracranial abnormalities or psychological disorders. Moreover, the psychiatrist did not elaborate about the assessment tools he used to rule out other psychiatric conditions, and was difficult to contact or reach as electronic records and shared health network are not established between different hospitals yet. Although the patient was referred for scaling and root planning (SRP), and was given oral hygiene instructions, a thorough periodontal evaluation and management were not carried out despite the increase susceptibility to periodontal disease noted in patients with psychiatric conditions due to poor oral hygiene or as a result of the use of xerogenic medications [21]. In addition to SRP, innovative and safe non-surgical periodontal approaches, such as the use paraprobiotics, can be explored as recent clinical trials concluded that paraprobiotics, in forms of toothpaste or mouth washes, reduces the oral periodontal pathogens load through various mechanisms [22]. Interestingly, in this case, the oral symptoms of DP had the “first to show and last to go” presentation pattern and was most recalcitrant to the psychiatric medication. The skin symptoms resolved faster than the oral and perioral symptoms after establishing the antipsychotic medication. To overcome challenges the OHCP might face with such patient, it is important to establish a positive OHCP–patient relationship. The patient may ask questions, such as “Do you think I'm crazy?” when suggesting a psychiatrist onboard, and the answer should be that not only because of the lack of evidence of any organic cause of the symptoms, but also to explain that psychiatrist might be able to prescribe medications that can help with the crawling sensation. OHCP can elaborate that some antidepressant medications, for example like doxepin, can be used as an antihistamine and can be of value to dermatologists. Future studies of similar nature should be directed toward preparing OM and orofacial pain specialist to recognize psychiatric conditions presenting as orofacial pain by using simple and effective screening tools, such as questionnaires, to be part of the history and management. Multidisciplinary approach is a key to manage such patients especially with psychiatrists to efficiently exchange information to maximize patient care.

## 4. Conclusion

OHCP should be familiar with the signs and symptoms of psychological disorder that can present as facial pain, and should have the skill to build rapport with such patients and to work closely with other specialists in a multidisciplinary approach to manage complex cases. Orofacial pain and OM specialists should be knowledgeable about medical conditions and medications that might induce symptoms of DP and should be able to exclude them before referring patients to a psychiatrist. In addition, OHCP should know about the oral symptoms that the patient may have as side effects of antipsychotics or other psychiatric medications. As in our case, overlaps between psychological conditions can appear and it might reflect the same pathophysiological process. Brief psychological assessment should be carried out at the OM clinic to identify patients with psychological conditions as it is important to address psychological disturbances in managing such patients.

## Figures and Tables

**Table 1:** Oral delusional parasitosis diagnosis and management: Summary.

Diagnosis	Management
History taking [9, 10]	Symptomatic treatment of oral lesions
Clinical examination [9–11]	Improve oral hygiene/periodontal health [21]
Laboratory testing [9–11]	Advise smoking cessation [21]
CBC/differential blood count	
Hb A1C	
TSH	
Liver function test	
Renal function test	
Serum vitamin B12	
Imaging: head CT/brain MRI to rule out intracranial abnormalities [17]	Refer for psychiatric evaluation: Pharmacological/non-pharmacological management [11, 13]
Psychiatric assessment tools: screening tools for depression/anxiety [9–11]	Refer for dermatology to improve aesthetic if needed
Biopsy if needed to rule out true parasitosis [19]	Follow-up regularly [21]

**Table 2:** Documented case reports of delusional parasitosis, Morgellons, and overlap syndrome.

Case report	Location	Year	Chief complaints	Significant medical history	Diagnosis	Management strategy
Maeda et al. [17]	Japan	1998	Threads emanating from the gingiva between teeth and then converting into worms	Left-side cerebral infarct (11 years ago)	Overlap syndrome	Antipsychotics
Hanihara et al. [18]	Japan	2009	Live worms infesting her mouth and moving around	Hematoma in the left posterior thalamic region revealed by CT	Delusional parasitosis	Sulpiride (atypical antipsychotic)
Dovigi [19]	USA	2010	Numerous fibers emerging from her oral lesion with itching and irritation	None	Morgellons	Not mentioned
Grosskopf et al. [20]	USA	2011	Hair and insects coming out of her maxillary gingiva with oral tickling sensation	Previous diagnosis of Morgellons	Overlap syndrome	Bupropion (atypical antidepressant) pimozide (atypical antipsychotic) CBT

## Data Availability

Data supporting this research article are available from the corresponding author or first author on reasonable request.
